# Juicebox.js Provides a Cloud-Based Visualization System for Hi-C Data

**DOI:** 10.1016/j.cels.2018.01.001

**Published:** 2018-02-07

**Authors:** James T. Robinson, Douglass Turner, Neva C. Durand, Helga Thorvaldsdóttir, Jill P. Mesirov, Erez Lieberman Aiden

**Affiliations:** 1 School of Medicine, University of California San Diego, La Jolla, CA 92093, USA; 2 Broad Institute of MIT and Harvard, Cambridge, MA 02142, USA; 3 The Center for Genome Architecture, Department of Molecular and Human Genetics, Baylor College of Medicine, Houston, TX 77030, USA; 4 Center for Theoretical Biological Physics, Rice University, Houston, TX 77030, USA; 5 Department of Computer Science, Rice University, Houston, TX 77030, USA; 6 Moores Cancer Center, University of California San Diego, La Jolla, CA 92037, USA

## Abstract

Contact mapping experiments such as Hi-C explore how genomes fold in 3D. Here, we introduce Juicebox.js, a cloud-based web application for exploring the resulting datasets. Like the original Juicebox application, Juicebox.js allows users to zoom in and out of such datasets using an interface similar to Google Earth. Juicebox.js also has many features designed to facilitate data reproducibility and sharing. Furthermore, Juicebox.js encodes the exact state of the browser in a shareable URL. Creating a public browser for a new Hi-C dataset does not require coding and can be accomplished in under a minute. The web app also makes it possible to create interactive figures online that can complement or replace ordinary journal figures. When combined with Juicer, this makes the entire process of data analysis transparent, insofar as every step from raw reads to published figure is publicly available as open source code.

## Graphical abstract

**In Brief:** Contact mapping experiments such as Hi-C explore how genomes fold in 3D. Here, we introduce Juicebox.js, a cloud-based web application for exploring and sharing the resulting datasets. Users can create sharable visualizations of their own datasets in a few minutes using cloud storage providers such as Dropbox, Google, and Amazon without coding.

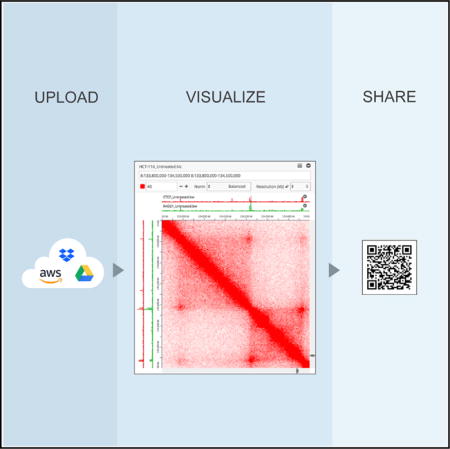

Hi-C and other contact mapping experiments measure the frequency of physical contact between loci in the genome. The resulting dataset, called a “contact map,” is often represented using a two-dimensional heatmap where the intensity of each pixel indicates the frequency of contact between a pair of loci. The highest resolution Hi-C heatmaps presently available contain trillions of pixels and exhibit structures across a wide range of size scales. To explore such data, we recently developed Juicebox (Durand et al., 2016a), a desktop application inspired by Google Earth. Juicebox enables users to interactively zoom in and out of Hi-C datasets and to perform many other functions.

One important use case for any scientific data visualization system is to enable multiple research groups to easily recapitulate a finding, thereby ensuring greater reproducibility of the scientific record. A web-based application enabling the visualization of Hi-C contact maps would make it easier to communicate findings compared with a desktop application. In fact, the original paper on Hi-C included such a web application (Lieberman-Aiden et al., 2009), but it only displayed data at very coarse resolution, suitable for the low-quality datasets that were available at that time. Although several other web-based Hi-C contact map browsers have been developed (Wang et al., 2017; Yardımcı and Noble, 2017), none of the tools that have been published to date support interactive zooming in real time, a critical feature that is essential to seamless exploration of the high resolution Hi-C data that are currently available. In addition, none of the extant tools can be easily extended with new data. (Although interested readers should see a recent preprint that also seeks to address these issues; Kerpedjiev et al., 2017).

Here, we present Juicebox.js, v1.0, a web application that implements many core features of the Juicebox desktop application (Figure 1). Juicebox.js enables users to load one or more contact maps, to zoom in and out, and to compare the contact maps with genomic tracks and 2D annotations. (Although these core features are present, users should be aware that the Juicebox desktop application currently supports many advanced features and views that are not available on Juicebox.js.) Juicebox.js supports both desktop and mobile devices; users can explore the maps using a keyboard and mouse, or by means of touch-screen gestures (such as pinch zoom).

Juicebox.js is designed to work with data in the *hic* format (Durand et al., 2016a), a compressed indexed format that enables fast queries and can be used with a wide variety of contact mapping experiments, such as dilution Hi-C (Lieberman-Aiden et al., 2009), *in situ* Hi-C (Rao et al., 2014), single-cell Hi-C (Nagano et al., 2013), Hi-C^2^ (Sanborn et al., 2015), ChIA-PET (Fullwood et al., 2009), HiChIP (Mumbach et al., 2016), and SPRITE (Quinodoz et al., 2017). This format has been adopted by many groups and several large consortia, including the Encyclopedia of DNA Elements (ENCODE) and the 4D Nucleome Consortium.

Users of Juicebox.js can load a *hic* file from their local hard disk or file system. However, because *hic* files encode billions of contacts, they can be very large (hundreds of gigabytes) and keeping local copies is often unwieldy. To address this need, Juicebox.js can access *hic* files located at an arbitrary URL, downloading only the small portion of the file that is required to fulfill each user request. Users can also remotely access genomic tracks (such as chromatin immunoprecipitation sequencing data) and 2D annotations (such as Hi-C loop calls) in all standard formats (*BigWig*, *bed*, *bedpe*, etc.) via URL. Loading via URL works with any standard file server, and with a range of cloud storage providers such as Amazon S3, Dropbox, and Google Drive. As an example, Juicebox.js is designed to automatically connect to ENCODE servers and remotely load any dataset generated by the consortium.

Juicebox.js has many features to facilitate reproducibility and sharing.

First, the complete state of any Juicebox.js instance can be encoded via a sharable URL link. (Note that files that were loaded from the local hard disk must be excluded due to intrinsic limitations that affect all browser-based apps.) The exact same fully interactive state can be recapitulated merely by opening this URL in another browser (including mobile browsers). The URL, which does not expire, can also be shared with the scientific public in a journal article, using social media such as Twitter, or even by means of a QR code. Sharable URLs can be created directly from Juicebox or programmatically by creating a URL text string with the necessary parameters included (such as the files to be accessed, genomic location, and color scale). For instance, a user could write a script to create a large number of URLs corresponding to a large number of features that have been identified in a given Hi-C dataset. Any of these features could then be explored further simply by clicking the corresponding link. Juicebox.js also supports oAuth 2.0, enabling password protection for sensitive datasets (https://oauth.net/2/).

Second, Juicebox.js instances, in any desired state, can be embedded in a webpage using a few lines of code. This makes it simple to include Juicebox.js on a lab website, a blog, in a news article, or in an online journal article. By embedding one or more Juicebox.js instances on a single webpage, it is possible to take a static publication figure and create an online version in which all contact maps are fully interactive. This enables other researchers to explore the results by changing the color scale and other display properties, and to ensure that an image is representative of the dataset as a whole, rather than a cherry-picked example. For a recent publication, we used Juicebox.js to create five interactive figures, which the journal was able to host alongside the online version of the paper (Rao et al., 2017) (see Table S1.)

Finally, Juicebox.js is a purely client-side application: it reads data directly from files hosted on standard web servers, but no application code is required on the server. Consequently, Juicebox.js can easily support an almost unlimited number of users. This also makes it possible to create interactive browsers with Juicebox.js for one or more *hic* files without writing a single line of code. For instance, a user can simply upload a *hic* file to Dropbox; use Dropbox to create a link to the file; and then load that link into Juicebox.js. The resulting Juicebox.js URL can immediately be shared with users all over the world, enabling them to interactively explore the data located on the Dropbox folder.

There is a great need to enhance the reproducibility of preclinical studies in biology and biomedicine (Collins and Tabak, 2014). For computational studies, sources of irreproducibility include factors such as the complexity of analyses and the temptation to speculate about ambiguous data (Collins and Tabak, 2014; Rowley and Corces, 2016). Such factors can be counteracted by making every step in an analysis maximally transparent, from the raw data to the published figure. Combined with the Juicer pipeline (Durand et al., 2016b), Juicebox.js makes this level of transparency and reproducibility achievable for Hi-C and other contact mapping experiments.

An instance of Juicebox.js is available at aidenlab.org/juicebox. The code, which is available at github.com/igvteam/juicebox.js, is open source and is licensed under the MIT license. Documentation for Juicebox.js is available at https://igvteam.github.io/juicebox.js. The test procedure and datasets associated with this publication are available at https://data.mendeley.com/archiver/fbgc85km6j.

## STAR★METHODS

Detailed methods are provided in the online version of this paper and include the following:

### KEY RESOURCES TABLE

**Table:** 

REAGENT or RESOURCE	SOURCE	IDENTIFIER
Software and Algorithms		
Juicebox.js instance	This paper; Mendeley Data	aidenlab.org/juicebox
Juicebox.js embeddable component	This paper	github.com/igvteam/juicebox.js
igv.js	N/A	github.com/igvteam/igv.js

### CONTACT FOR REAGENT AND RESOURCE SHARING

Further information and requests for resources and reagents should be directed to and will be fulfilled by the Lead Contact, Erez Lieberman Aiden (erez@erez.com).

### DATA AND SOFTWARE AVAILABILITY

Instance of Juicebox.js: aidenlab.org/juicebox

Juicebox.js source code: github.com/igvteam/juicebox.js

Juicebox.js documentation: igvteam.github.io/juicebox.js

Test procedure & datasets with this publication: https://data.mendeley.com/archiver/fbgc85km6j

### METHOD DETAILS

Only a web browser and internet connection is required to run Juicebox.js at aidenlab.org/juicebox.

Embedding the Juicebox.js component in web pages depends on igv.js. The Developer Documentation pages at igvteam.github.io/juicebox.js has more information. See also www.igv.org for igv.js information.

## Supplementary Material

supplement

## Figures and Tables

**Figure 1:**
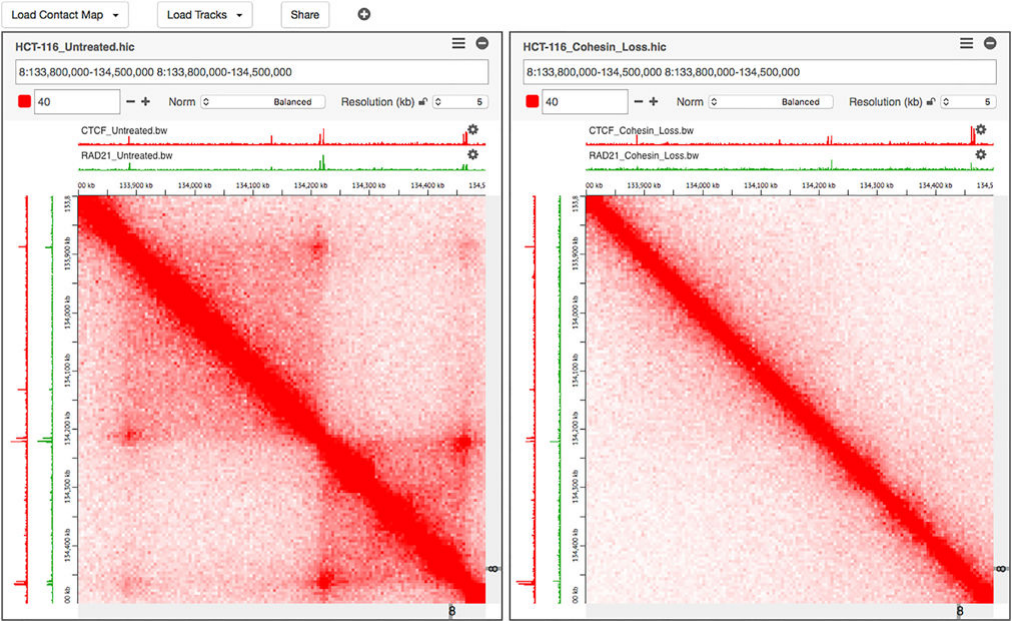
Juicebox.js Makes It Easy to Share Interactive Visualizations of Contact Mapping Data Derived from Hi-C and Other Experiments Hi-C maps from new experiments can be easily added and juxtaposed with tracks from ENCODE and other sources. It is possible to zoom in and out in real time using either a mouse or touch-screen gestures. Display parameters such as the color scale and the normalization can be adjusted interactively. The complete state of the browser can always be encoded as a sharable URL. No programming is necessary to share and explore new datasets. Left: A loop resolution Hi-C map showing all contacts within an 700 kb genomic interval, generated using HCT-116 human colorectal carcinoma cells. Loops, which form here due to physical tethering between two CTCF- and cohesin-bound loci, manifest as bright peaks away from the diagonal. Contact domains, genomic intervals that exhibit enhanced contact frequency within themselves, manifest as bright squares along the diagonal. When the two anchors of a loop demarcate a contact domain, the resulting feature is called a “loop domain.” Right: The same region in HCT-116 cells after the RAD21 subunit of the cohesin complex has been degraded using an auxin-inducible degron system. The loop domains all disappear completely, demonstrating that they are dependent on cohesin. An interactive version of this figure is available at https://aidenlab.org/figures/Robinson-Cell-Systems-2017/figure1.html (see also Table S1).

## Highlights

- Juicebox.js enables users to explore Hi-C contact maps from their web browser
- The exact state of the browser can be encoded in a sharable URL
- Users can create sharable maps of their own data in a few minutes

## References

[R1] Collins FS, Tabak LA (2014). Policy: NIH plans to enhance reproducibility. Nature.

[R2] Durand NC, Robinson JT, Shamim MS, Machol I, Mesirov JP, Lander ES, Aiden EL (2016a). Juicebox provides a visualization system for hi-c contact maps with unlimited zoom. Cell Syst.

[R3] Durand NC, Shamim MS, Machol I, Rao SS, Huntley MH, Lander ES, Aiden EL (2016b). Juicer provides a one-click system for analyzing loop-resolution Hi-C experiments. Cell Syst.

[R4] Fullwood MJ, Liu MH, Pan YF, Liu J, Xu H, Mohamed YB, Orlov YL, Velkov S, Ho A, Mei PH (2009). Anoestrogen-receptor-alpha-bound human chromatin interactome. Nature.

[R5] Kerpedjiev P, Abdennur N, Lekschas F, McCallum C, Dinkla K, Strobelt H, Luber JM, Ouellette SB, Ahzir A, Kumar N (2017). HiGlass: web-based visual comparison and exploration of genome interaction maps. bioRxiv.

[R6] Lieberman-Aiden E, van Berkum NL, Williams L, Imakaev M, Ragoczy T, Telling A, Amit I, Lajoie BR, Sabo PJ, Dorschner MO (2009). Comprehensive mapping of long-range interactions reveals folding principles of the human genome. Science.

[R7] Mumbach MR, Rubin AJ, Flynn RA, Dai C, Khavari PA, Greenleaf WJ, Chang HY (2016). HiChIP: efficient and sensitive analysis of protein-directed genome architecture. Nat Methods.

[R8] Nagano T, Lubling Y, Stevens TJ, Schoenfelder S, Yaffe E, Dean W, Laue ED, Tanay A, Fraser P (2013). Single-cell Hi-C reveals cell-to-cell variability in chromosome structure. Nature.

[R9] Quinodoz SA, Ollikainen N, Tabak B, Palla A, Schmidt JM, Detmar E, Lai M, Shishkin A, Bhat P, Trinh V (2017). Higher-order inter-chromosomal hubs shape 3-dimensional genome organization in the nucleus. bioRxiv.

[R10] Rao SS, Huntley MH, Durand NC, Stamenova EK, Bochkov ID, Robinson JT, Sanborn AL, Machol I, Omer AD, Lander ES, Aiden EL (2014). A 3D map of the human genome at kilobase resolution reveals principles of chromatin looping. Cell.

[R11] Rao SSP, Huang SC, Glenn St Hilaire B, Engreitz JM, Perez EM, Kieffer-Kwon KR, Sanborn AL, Johnstone SE, Bascom GD, Bochkov ID (2017). Cohesin loss eliminates all loop domains. Cell.

[R12] Rowley MJ, Corces VG (2016). Minute-made data analysis: tools for rapid interrogation of Hi-C contacts. Mol Cell.

[R13] Sanborn AL, Rao SS, Huang SC, Durand NC, Huntley MH, Jewett AI, Bochkov ID, Chinnappan D, Cutkosky A, Li J (2015). Chromatin extrusion explains key features of loop and domain formation in wild-type and engineered genomes. Proc Natl Acad Sci USA.

[R14] Wang Y, Zhang B, Zhang L, An L, Xu J, Li D, Choudhary MNK, Li Y, Hu M, Hardison R (2017). The 3D Genome Browser: a web-based browser for visualizing 3D genome organization and long-range chromatin interactions. bioRxiv.

[R15] Yardımcı GG, Noble WS (2017). Software tools for visualizing Hi-C data. Genome Biol.

