# Supplementary material for: Juicebox.js Provides a Cloud-Based Visualization System for Hi-C Data
Source: Cell Syst. Author manuscript; Available in PMC 2018 Jul 16. (PMC6047755; doi:10.1016/j.cels.2018.01.001)
Supplement: supplement [file NIHMS949176-supplement.pdf]

Cell Systems, Volume *6*

Supplemental Information

Juicebox.js Provides a Cloud-Based

Visualization System for Hi-C Data

James T. Robinson, Douglass Turner, Neva C. Durand, Helga Thorvaldsdóttir, Jill P. Mesirov, and Erez Lieberman Aiden

Table S1. Interactive Figures from Rao et al., Cell 2017, "Cohesin Loss Eliminates All Loop Domains". Related to Figure 1.

Figure #	Figure Title	URL
Figure 2	Cohesin Degradation Eliminates Loop Domains.	http://www.cell.com/cell/9802-figure-2
Figure 3	Genome Compartmentalization Is Strengthened after Cohesin Degradation	http://www.cell.com/cell/9802-figure-3
Figure 4	Cohesin Loss Causes Superenhancers to Co-localize, Forming Hundreds of Links within and across Chromosomes	http://www.cell.com/cell/9802-figure-4
Figure 5	In the Absence of Cohesin, a Clique Spanning More Than 20 Superenhancers Forms Pairwise Links and Higher-Order Hubs	http://www.cell.com/cell/9802-figure-5
Figure 6	Molecular Dynamics Simulations Combining Extrusion and Compartmentalization Can Recapitulate Hi-C Experimental Results	http://www.cell.com/cell/9802-figure-6
Figure 7	Cohesin Degradation Results in Strong Down-Regulation of Genes near Superenhancers but Does Not Result in Widespread Ectopic Gene Activation	http://www.cell.com/cell/9802-figure-7
